# Predicting survival in cancer patients with and without 30‐day readmission of an unplanned hospitalization using a deficit accumulation approach

**DOI:** 10.1002/cam4.2472

**Published:** 2019-09-07

**Authors:** Timothy N. Hembree, Sarah Thirlwell, Richard R. Reich, Smitha Pabbathi, Martine Extermann, Asha Ramsakal

**Affiliations:** ^1^ Department of Internal and Hospital Medicine H. Lee Moffitt Cancer Center and Research Institute Tampa Florida; ^2^ Department of Supportive Care Medicine H. Lee Moffitt Cancer Center and Research Institute Tampa Florida; ^3^ Biostatistics Core H. Lee Moffitt Cancer Center and Research Institute Tampa Florida; ^4^ Senior Adult Oncology Program H. Lee Moffitt Cancer Center and Research Institute Tampa Florida

**Keywords:** cancer, clinical documentation, deficit‐accumulation index, late‐stage cancer, survival

## Abstract

**Background:**

For cancer patients with an unplanned hospitalization, estimating survival has been limited. We examined factors predicting survival and investigated the concept of using a deficit‐accumulation survival index (DASI) in this population.

**Methods:**

Data were abstracted from medical records of 145 patients who had an unplanned 30‐day readmission between 01/01/16 and 09/30/16. Comparison data were obtained for patients who were admitted as close in time to the date of index admission of a study patient, but who did not experience a readmission within 30 days of their discharge date. Our survival analysis compared those readmitted within 30 days versus those who were not. Scores from 23 medical record elements used in our DASI system categorized patients into low‐, moderate‐, and high‐score groups.

**Results:**

Thirty‐day readmission was strongly associated with the survival (adjusted hazard ratio [HR] 2.39; 95% confidence interval [CI], 1.46‐3.92). Patients readmitted within 30 days of discharge from index admission had a median survival of 147 days (95% CI, 85‐207) versus patients not readmitted who had not reached median survival by the end of the study (*P *< .0001). DASI was useful in predicting the survival; median survival time was 78 days (95% CI, 61‐131) for the high score, 318 days (95% CI, 207‐426) for the moderate score, and not reached as of 426 days (95% CI, 251 to undetermined) for the low‐score DASI group (*P *< .0001).

**Conclusions:**

Patients readmitted within 30 days of an unplanned hospitalization are at higher risk of mortality than those not readmitted. A novel DASI developed from clinical documentation may help to predict survival in this population.

## Graphical abstract

Cancer patients readmitted within 30 days of an unplanned hospitalization are at a higher risk of mortality. A novel index developed from clinical documentation may help to predict survival in this population.

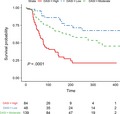

## 1 INTRODUCTION

Several factors have been shown to influence survival in newly diagnosed cancer patients, including variables related to the tumor (size, grade, and biological characteristics, including molecular alterations), tumor stage, and those related to the nature and quality of treatment.^1^ Although these factors are useful in estimating survival and for treatment planning in the initial phase after diagnosis, these tend to be less helpful when it comes to predicting short‐term outcomes in advanced states of disease. Advanced cancer is defined as cancer that is unlikely to be cured or controlled with treatment (NCI dictionary) and treatment is primarily palliative to control symptoms. Common symptoms of advanced cancer include pain, nausea, and loss of appetite, tiredness, and breathlessness.^2^ This study is designed to help identify objective measures/factors that discriminate for more advanced disease presentations and can help to define the terminal phase of cancer. If we can define the terminal phase more objectively, we can communicate the prognosis more clearly and plan our treatment approach in accordance with the patient's wishes.

In regards to survival, individual cancer patients exhibit a remarkable amount of variation in outcomes. To understand this variation, researchers have attempted to identify clinical factors that can be used to predict the prognosis of individual cancer patients. The magnitude of this work is staggering with one literature review^3^ identifying 887 publications documenting 169 clinical, laboratory, and molecular prognostic factors in NSCLC patients. Despite the abundance of studies, results conflict on the importance of certain markers in predicting outcomes.^4^ Hence, only a few prognostic factors, such as extent of disease and performance status have been widely accepted and are used in clinical practice and defining the terminal phase in advanced cancer patients remains an ongoing challenge.

In the terminal phase, the clinical course of most cancers is fairly universal, with indicators of terminal disease, such as failing performance status, advanced age, weight loss, anorexia, hypercalcemia, malnutrition, and laboratory abnormalities, indicating widespread inflammation or extensive disease. Survival rates for these presentations have not significantly changed over the past 30 years, regardless of cancer type.^5^

Cancer‐related morbidity and treatment‐related complications often result in a decompensation in which the cancer patient requires hospitalization. The average cancer patient spends about 5 weeks in the hospital during the last 6 months of life, and the terminal phase of the disease is associated with an exponential rise in hospitalization rate.^6^ Time trends in the rate of hospitalization of cancer patients over the last 6 months of life show that the proportion of patients hospitalized on any given day increases slowly, until approximately 3 months before death, but then increases at an accelerating rate as death approaches.^7^ Other more recent studies show that aggressiveness in end‐of‐life care for cancer patients is on the rise, which translates to more frequent and repeated hospital admissions, emergency department visits, and intensive care unit admissions.^8^ Another study reported that hospitalization may be underestimated in patients with advanced cancers. In patients with advanced cancer at the time of diagnosis (defined as stage IV for all cancers, stage IIIB for non‐small‐cell lung cancer, and stage III for pancreatic cancer), 71% were hospitalized at least once during the first year after diagnosis and 16% were hospitalized three times or more.^9^

We believe that the first unplanned hospitalization event may be a harbinger that signals the transition to the terminal phase and a 30‐day readmission event signifies an even stronger decline in overall ability to respond to stress and necessitates a re‐evaluation of survival at the time of hospitalization. Most studies in cancer patients have attempted to model a limited number of factors to predict survival, and the prognostic models have been developed using poor methods which compromises the reliability and clinical relevance of the models, prognostic indices, and risk groups derived from them.^10^ This study will examine survival as a function of accumulated deficits—the index created will be used as a proxy measure of vulnerability to poor outcomes/mortality. As patients experience cancer‐related morbidity or treatment‐related complications requiring hospitalization, we believe that their survival is a function of accumulated deficits. The Rockwood method is a model based on the accumulation of deficits that proposes that as health deficits increase so do the risk of adverse outcomes.^11^ The concept that risk is related to deficit accumulation extends to a range of illnesses, including dementia,^12^ osteoporosis,^13^ and coronary heart disease.^14^ In these studies, deficit accumulation indicates increased disease expression and mortality risk. Deficits can be those outside of known risk factors for the disease and can include general health deficits and deficits related to special clinical populations and specific procedures. Laboratory tests and biomarkers have also been used as deficit indicators associated with risk of specific events.^15^, ^16^ An advantage to the deficit accumulation approach is that it allows multiple small effects, including ones that individually are not significantly associated with the outcome of interest, to add up. A deficit‐accumulation index to assess frailty in older patients undergoing chemotherapy was shown to be useful in predicting likelihood of experiencing chemotherapy‐related toxicities.^17^

We evaluated 30‐day hospital readmission and accumulated deficits as predictors of survival in cancer patients with unanticipated hospital admissions. The DASI components, as listed in Table 1, were chosen based on literature review of known prognostic indicators and used together in accordance with the Rockwood Methodology.^11^ The factors chosen for the deficit‐accumulation survival index (DASI) included known prognostic indicators of cancer survival in the terminal cancer patient: markers of malnutrition (prognostic nutrition index [PNI],^18^ weight,^19^ and albumin level^20^); inflammatory indices (neutrophil‐to‐lymphocyte ratio [NLR],^21^, ^22^, ^23^ platelet‐to‐lymphocyte ratio [PLR],^24^, ^25^, ^26^ and systemic immune‐inflammation index [SII]^27^); and biological factors (leukocytosis,^28^ hypercalcemia,^29^ lymphopenia,^30^ renal insufficiency,^31^, ^32^ and hyponatremia^33^). Other factors of interest included type of cancer,^34^ health care utilization in the 6 months before the index admission, 30‐day readmission, length of index admission, presence of polypharmacy,^35^, ^36^ marital status as proxy for social support,^37^ fall risk,^38^ reason for index admission, reason for 30‐day readmission, Eastern Cooperative Oncology Group (ECOG) performance status (PS),^39^ and metastatic status.

**Table 1:** Twenty‐three factors and cutoff levels utilized for the deficit‐accumulation

Factor/cutoff level	Deficit
Readmission	
Yes	1
No	0
Marital status/support	
Single/divorced/widowed	1
Married	0
Language	
Not english	1
English	0
Fall risk	
High	1
Moderate/low	0
Health care utilization during prior 6 months (count visits)	
>2	1
<2	0
BMI (kg/m^2^)	
<19	1
>19	0
Calcium corrected (mg/dL)	
≥11	1
<11	0
Creatinine (mg/dL)	
>1.3	1
<1.3	0
ECOG performance status	
2, 3, 4	1
0, 1	0
NLR (index)	
>5	1
<5	0
Neutrophils (k/μL)	
<1.8	1
>1.8	0
PLR (index)	
>250	1
<250	0
Platelet count (k/μL)	
>450 or <150	1
150‐450	0
Hemoglobin (g/dL)	
<12	1
>12	0
Length of index admission (days)	
>5	1
<5	0
Lymphocytes (k/μL)	
<1.1	1
>1.1	0
SII (index)	
>1600	1
<1600	0
Albumin (g/dL)	
<3.5	1
>3.5	0
Sodium (mmol/L)	
<135	1
>135	0
WBC (k/μL)	
>11	1
<11	0
Number of medications (count)	
>5	1
<5	0
PNI (index)	
<45	1
>45	0
Metastasis	
Yes	1
No	0

Abbreviations: BMI, body mass index; ECOG, Eastern Cooperative Oncology Group; NLR, neutrophil‐to‐lymphocyte ratio; PNI, prognostic nutrition index; PLR, platelet‐to‐lymphocyte ratio; SII, systemic immune‐inflammation index; WBC, white cell blood count.

## 2 PATIENTS AND METHODS

In this retrospective cohort study design, we identified patients who were admitted to the Internal Hospital Medicine (IHM) service at Moffitt Cancer Center between 01/01/16 and 09/30/16 with unplanned readmissions within 30 days of discharge from an index admission. To identify a control group, we ascertained the admission date of each study patient's index admission, which ranged from 12/26/15 to 09/14/16. A specific control patient who did not experience a readmission within 30 days of discharge was chosen as a match based on the proximity of his/her first day of admission to the date and time of a study patient's first day of admission. We identified 148 pairs of patients. On review, a few were not evaluable, resulting in 132 unplanned hospitalized patients (controls) and 139 patients with a readmission within 30‐day discharge. The last date of follow‐up/survival information was 4/13/2017.

Our center is a free‐standing comprehensive cancer center focused solely on the care of patients with a cancer diagnosis and their treatment. The Internal Hospital Medicine service admits patients for active cancer treatment, complications of cancer treatment, and morbidity associated with cancer. Patients with malignant hematologic diseases that have medical issues are rarely admitted to our service. For the purpose of this study, those with planned admissions were excluded from the 30‐day readmission cohort.

We collected the following clinical data from patient electronic medical records: length of stay of index admission, occurrence of a 30‐day readmission, days between readmission and discharge from index admission, age, marital status, language, reason for index admission, diagnosis(es) related to index admission, number of medications (scheduled and as needed) at discharge from index admission, number of admissions to our center in the 6 months before index admission, number of admissions to our center's intensive care unit in the 6 months before index admission, number of urgent care visits to our center's direct referral center in the 6 months before index admission, Morse fall risk level at time of index admission, weight, height, primary tumor site, number, and location of metastatic sites, ECOG PS, white blood cell count, hemoglobin level, platelet count, neutrophil count, lymphocyte count, serum sodium level, serum creatinine level, serum corrected calcium level, and serum albumin level.

We calculated NLR by dividing the number of neutrophils by the number of lymphocytes, the PLR by dividing the number of platelets by the number of lymphocytes, the SII by multiplying the PLR by the number of neutrophils, and the PNI by multiplying the albumin level by number of lymphocytes.

The reason for admission variable (chief complaint) was determined by review of patient history and physical assessment at time of admission. If there were multiple similar reasons for admission (chief complaints), these were then grouped for statistical analysis. The groupings are as follows: (a) pain (back pain, neoplasm‐related pain, abdominal pain); (b) neurologic complaints (syncope, dizziness, headache, diplopia, ataxia); (c) dyspnea (shortness of breath, hypoxia); (d) adverse effects of chemotherapy (nausea, vomiting, diarrhea within 1 week of chemotherapy); (e) abnormal findings on electrocardiogram, laboratory values, or radiographic imaging (combined group); and (f) other (any “reason” with count <10). Regarding the variable of “cancer type,” for any type with a count of <10, it was grouped into the category of “other.”

The outcome of interest was survival. The 23 factors used for DASI calculation and cutoff levels are shown in Table 1. This study was approved by the Chesapeake Institutional Review Board, which met the criteria for waiver of consent and of Health Insurance Portability and Accountability Act authorization.

### 2.1 Statistical analyses

One goal of this analysis was to examine the relation between hospital readmission in cancer patients and overall survival. A Cox proportional hazards model tested time from initial hospital admission until death, with censoring at last follow‐up for living patients. A hazard ratio (HR) with a 95% confidence interval (CI) described the relation between hospital readmission and survival, with Kaplan‐Meier curves used to depict this relation. Also of interest was whether this relation remained statistically significant while accounting for other demographic (e.g. sex) and clinical (e.g. type of cancer) variables.

Univariate models were tested to determine which variables should be included in a single multivariable Cox model (*P* < .05). NLR, neutrophil, PLR, platelet, and SII were all highly correlated; hence, only NLR was carried into multivariable analysis. Our multivariable model required the inclusion of cancer type, regardless of its statistical significance, to demonstrate that, in the terminal stage, the type of cancer does not play a prominent role in time to death. We also planned to include recent health care utilization regardless in the multivariable model. The outcome of interest in the multivariable model was the relation between hospital readmission and survival. With 271 patients, we were able to include as many as 13 covariates with minimal concern for model overfitting.^40^ We ended up including nine variables in the model.

Another goal of this study was to use an accumulation of deficits approach to predict survival. Deficits were summed across 23 factors and divided by the number of factors to create the DASI. DASI scores were then used to categorize patients into 3 groups: low (cutoff value of <0.30), moderate (cutoff value of 0.31‐0.48), and high (cutoff value of >0.48).

We used the Cox proportional hazards model and HR and 95% CI to describe the relation between deficit score and survival. A Kaplan‐Meier curve depicted this relation for the low, medium, and high deficit score groups. Data were analyzed using SAS 9.4 (SAS Institute Inc, Cary, NC).

With 271 patients, we had 80% statistical power to detect a hazard ratio of 1.54 or greater for the readmission outcome, and 1.69 or greater for the DASI outcome (assuming a 2‐sided test with a nominal alpha of 0.05).

## 3 RESULTS

Table 2 shows characteristics of the 271 patients. The median age of patients was 63 years (range, 19‐92 years). Most patients were white (86%), English speaking (96%), and married (68%), with 51% male and 49% female.

**Table 2:** Descriptive analysis

Variable	Level	N = 266	%
Readmission (Y/N)	N	132	49.6
	Y	134	50.4
Sex	Female	125	47.0
	Male	141	53.0
Marital status	Divorced	27	10.3
	Married	180	69.0
	Single	33	12.6
	Widow	21	8.0
	Missing	5	—
Language	English	255	95.9
	Not English	11	4.1
Race	Not White	39	14.7
	White	227	85.3
Categorized Morse fall risk	High	103	39.3
	Low	2	0.8
	Moderate	148	56.5
	Universal	9	3.4
	Missing	4	—
ECOG	0	26	15.4
	1	105	62.1
	2	25	14.8
	3	13	7.7
	Missing	97	—
General readmission reason category	Cancer related	64	24.1
	General medical	95	35.7
	Treatment related	32	12.0
	Uncontrolled Sx	75	28.2
Histology category	Carcinoma	195	73.6
	Melanoma	20	7.5
	Other	30	11.3
	Sarcoma	20	7.5
	Missing	1	—
Vital status	Alive	157	59.0
	Dead	109	41.0
Metastasis	0	99	38.4
	1	159	61.6
	Missing	8	—
Reason for admission	Abnormal EKG/Lab/Image	26	9.8
	Chemotherapy side effect	31	11.7
	Dehydration	11	4.1
	Dyspnea	40	15.0
	Fever	26	9.8
	Neurological Complaint	10	3.8
	Other	63	23.7
	Pain	59	22.2
Cancer type	Bladder	10	3.8
	Breast	19	7.1
	GI	61	22.9
	Head and neck	13	4.9
	Hepatocellular	7	2.6
	Lung	48	18.0
	Melanoma	21	7.9
	Neuroendocrine	8	3.0
	Other	65	24.4
	Sarcoma	14	5.3
Clinic	Breast	19	7.1
	Cutaneous	25	9.4
	Endocrine	3	1.1
	GI	86	32.3
	GU	29	10.9
	Gyn	2	0.8
	Head and neck	18	6.8
	Malignant heme	14	5.3
	Neuro‐onc	2	0.8
	Sarcoma	19	7.1
	Thoracic	49	18.4
Age	Mean	61.63	—
	Median	63	—
	Minimum	19	—
	Maximum	87	—
	SD	13.05	—
	Missing	0	—
Health care visits previous 6 months	Mean	0.65	—
	Median	0	—
	Minimum	0	—
	Maximum	6	—
	SD	0.97	—
	Missing	0	—
BMI	Mean	26.50	—
	Median	25.86	—
	Minimum	14.76	—
	Maximum	57.15	—
	SD	6.39	—
	Missing	40	—
Corrected calcium	Mean	9.57	—
	Median	9.50	—
	Minimum	7.50	—
	Maximum	16	—
	SD	0.91	—
	Missing	28	—
Creatinine	Mean	1.02	—
	Median	0.80	—
	Minimum	0.20	—
	Maximum	7	—
	SD	0.77	—
	Missing	16	—
Days between current admission and index admission	Mean	11.06	—
	Median	10	—
	Minimum	1	—
	Maximum	27	—
	SD	7.54	—
	Missing	—	—
Karnofsky	Mean	77.39	—
	Median	80	—
	Minimum	40	—
	Maximum	100	—
	SD	12.53	—
	Missing	124	—
Karnofsky score converted to ECOG	Mean	0.87	—
	Median	1	—
	Minimum	0	—
	Maximum	3	—
	SD	0.64	—
	Missing	124	—
Difference between ECOG score and Karnofsky conversion	Mean	0.29	—
	Median	0	—
	Minimum	−2	—
	Maximum	3	—
	SD	0.82	—
	Missing	188	—
Height(cm)	Mean	169	—
	Median	169	—
	Minimum	145	—
	Maximum	198	—
	SD	9.87	—
	Missing	34	—
Hemoglobin	Mean	10.68	—
	Median	10.70	—
	Minimum	2	—
	Maximum	18.70	—
	SD	2.33	—
	Missing	5	—
Length of index admission (days)	Mean	4.88	—
	Median	3	—
	Minimum	0	—
	Maximum	63	—
	SD	5.89	—
	Missing	0	—
Lymphocytes	Mean	1.59	—
	Median	0.87	—
	Minimum	0.080	—
	Maximum	104	—
	SD	7.16	—
	Missing	18	—
Morse fall risk	Mean	47.47	—
	Median	45	—
	Minimum	0	—
	Maximum	100	—
	SD	18.11	—
	Missing	3	—
NLR	Mean	10.36	—
	Median	7.16	—
	Minimum	0.010	—
	Maximum	137	—
	SD	13.56	—
	Missing	46	—
Neutrophil	Mean	7.07	—
	Median	5.73	—
	Minimum	0.010	—
	Maximum	33.24	—
	SD	4.77	—
	Missing	44	—
PLR	Mean	359	—
	Median	253	—
	Minimum	1.54	—
	Maximum	3089	—
	SD	370	—
	Missing	20	—
Platelet Count	Mean	239	—
	Median	214	—
	Minimum	1	—
	Maximum	941	—
	SD	147	—
	Missing	5	—
SII	Mean	2546	—
	Median	1618	—
	Minimum	0.038	—
	Maximum	19 293	—
	SD	2938	—
	Missing	46	—
Albumin	Mean	3.42	—
	Median	3.40	—
	Minimum	0.30	—
	Maximum	7.40	—
	SD	0.71	—
	Missing	24	—
Sodium	Mean	136	—
	Median	136	—
	Minimum	116	—
	Maximum	157	—
	SD	4.98	—
	Missing	17	—
WBC	Mean	9.95	—
	Median	7.92	—
	Minimum	0.10	—
	Maximum	138	—
	SD	11.62	—
	Missing	5	—
Number of Medications on Admission	Mean	11.36	—
	Median	11	—
	Minimum	0	—
	Maximum	31	—
	SD	4.83	—
	Missing	4	—
PNI	Mean	42.53	—
	Median	39.05	—
	Minimum	6.85	—
	Maximum	549	—
	SD	38.05	—
	Missing	38	—

Abbreviations: BMI, body mass index; ECOG, Eastern Cooperative Oncology Group; NLR, neutrophilto‐lymphocyte ratio; PNI, prognostic nutrition index; PLR, platelet‐to‐lymphocyte ratio; SII, systemic immune‐inflammation index; WBC, white cell blood count.

Patients with gastrointestinal cancers (23%) and lung cancers (16%) were most commonly represented among those who had an unplanned admission to our hospital (Table 2). Greater than 61% had documented metastatic disease; most patients were admitted with a chief complaint associated with common symptoms of advanced cancer (pain (21%), chemotherapy side effect (11%), dyspnea (15%), or fever (8%). However, reason for index admission was not a statistically significant predictor of survival in multivariable analysis (see Table 5).

Table 3 compares each variable by 30‐day readmission. Few differences reached statistical significance (*P* < .05). The few differences included age, with readmitted patients being slightly younger. The readmitted patients also had higher health care visits, length of index admission, medications, and NLR. Not surprisingly, the DASI was higher in readmitted patients.

**Table 3:** Univariate comparison of all variables by cases and controls

Covariate	Statistics	Level	30‐d readmission (Y/N)		Parametric *P*‐value*
			Controls No N = 132	Cases Yes N = 134	
Sex	N (Col %)	Female	64 (48.48)	61 (45.52)	.628
	N (Col %)	Male	68 (51.52)	73 (54.48)	
Marital status	N (Col %)	Divorced	16 (12.21)	11 (8.46)	.122
	N (Col %)	Married	91 (69.47)	89 (68.46)	
	N (Col %)	Single	11 (8.4)	22 (16.92)	
	N (Col %)	Widow	13 (9.92)	8 (6.15)	
Language	N (Col %)	English	128 (96.97)	127 (94.78)	.369
	N (Col %)	Not English	4 (3.03)	7 (5.22)	
Race	N (Col %)	Not White	18 (13.64)	21 (15.67)	.639
	N (Col %)	White	114 (86.36)	113 (84.33)	
Categorized morse fall risk	N (Col %)	High	54 (40.91)	49 (37.69)	.154
	N (Col %)	Low	0 (0)	2 (1.54)	
	N (Col %)	Moderate	71 (53.79)	77 (59.23)	
	N (Col %)	Universal	7 (5.3)	2 (1.54)	
ECOG	N (Col %)	0	14 (17.72)	12 (13.33)	.156
	N (Col %)	1	50 (63.29)	55 (61.11)	
	N (Col %)	2	7 (8.86)	18 (20)	
	N (Col %)	3	8 (10.13)	5 (5.56)	
Admission reason	N (Col %)	Abnormal EKG/Lab/Image	9 (6.82)	17 (12.69)	.199
	N (Col %)	Chemotherapy side effect	14 (10.61)	17 (12.69)	
	N (Col %)	Dehydration	6 (4.55)	5 (3.73)	
	N (Col %)	Dyspnea	26 (19.7)	14 (10.45)	
	N (Col %)	Fever	10 (7.58)	16 (11.94)	
	N (Col %)	Neurological complaint	6 (4.55)	4 (2.99)	
	N (Col %)	Other	28 (21.21)	35 (26.12)	
	N (Col %)	Pain	33 (25)	26 (19.4)	
Cancer type	N (Col %)	Bladder	4 (3.03)	6 (4.48)	.532
	N (Col %)	Breast	10 (7.58)	9 (6.72)	
	N (Col %)	GI	38 (28.79)	23 (17.16)	
	N (Col %)	Head and Neck	5 (3.79)	8 (5.97)	
	N (Col %)	Hepatocellular	2 (1.52)	5 (3.73)	
	N (Col %)	Lung	22 (16.67)	26 (19.4)	
	N (Col %)	Melanoma	11 (8.33)	10 (7.46)	
	N (Col %)	Neuroendocrine	4 (3.03)	4 (2.99)	
	N (Col %)	Other	28 (21.21)	37 (27.61)	
	N (Col %)	Sarcoma	8 (6.06)	6 (4.48)	
Histology category	N (Col %)	Carcinoma	101 (77.1)	94 (70.15)	.583
	N (Col %)	Melanoma	8 (6.11)	12 (8.96)	
	N (Col %)	Other	14 (10.69)	16 (11.94)	
	N (Col %)	Sarcoma	8 (6.11)	12 (8.96)	
Clinic	N (Col %)	Breast	10 (7.58)	9 (6.72)	.920
	N (Col %)	Cutaneous	13 (9.85)	12 (8.96)	
	N (Col %)	Endocrine	1 (0.76)	2 (1.49)	
	N (Col %)	GI	46 (34.85)	40 (29.85)	
	N (Col %)	GU	12 (9.09)	17 (12.69)	
	N (Col %)	Gyn	0 (0)	2 (1.49)	
	N (Col %)	Head and Neck	9 (6.82)	9 (6.72)	
	N (Col %)	Malignant heme	8 (6.06)	6 (4.48)	
	N (Col %)	Neuro‐onc	1 (0.76)	1 (0.75)	
	N (Col %)	Sarcoma	10 (7.58)	9 (6.72)	
	N (Col %)	Thoracic	22 (16.67)	27 (20.15)	
DASI	N (Col %)	HIG	25 (18.94)	54 (40.3)	**<.001**
	N (Col %)	MOD	69 (52.27)	64 (47.76)	
	N (Col %)	LOW	38 (28.79)	16 (11.94)	
Metastasis	N (Col %)	0	49 (37.98)	50 (38.76)	.898
	N (Col %)	1	80 (62.02)	79 (61.24)	
Age	N		132	134	**.048**
	Mean		63.22	60.06	
	Median		65	63	
Health care visits previous 6 months	N		132	134	**.045**
	Mean		0.53	0.77	
	Median		0	0	
SCMC visits previous 6 months	N		132	134	.232
	Mean		0.25	0.42	
	Median		0	0	
Weight	N		124	133	.630
	Mean		77.47	76.24	
	Median		72.85	74.7	
Corrected Calcium	N		119	119	.258
	Mean		9.51	9.64	
	Median		9.5	9.5	
Creatinine	N		124	126	.959
	Mean		1.02	1.02	
	Median		0.8	0.8	
ECOG	N		79	90	.592
	Mean		1.11	1.18	
	Median		1	1	
Karnofsky	N		68	74	.568
	Mean		76.76	77.97	
	Median		80	80	
Hemoglobin	N		129	132	.868
	Mean		10.66	10.7	
	Median		10.8	10.65	
Length of index admission (days)	N		132	134	**<.001**
	Mean		3.61	6.12	
	Median		3	4	
Lymphocytes	N		119	129	.773
	Mean		1.45	1.71	
	Median		0.96	0.77	
NLR	N		112	108	**.018**
	Mean		8.23	12.56	
	Median		6.22	7.73	
Neutrophil	N		113	109	.132
	Mean		6.59	7.56	
	Median		5.5	6.35	
PLR	N		119	127	.858
	Mean		363.37	354.89	
	Median		250	268.24	
Platelet count	N		129	132	.598
	Mean		243.63	234.02	
	Median		208	218.5	
SII	N		112	108	.282
	Mean		2336.75	2763.63	
	Median		1400.96	2056.93	
Albumin	N		121	121	.119
	Mean		3.49	3.35	
	Median		3.5	3.4	
Sodium	N		124	125	.951
	Mean		135.86	135.82	
	Median		136	136	
WBC	N		129	132	.952
	Mean		9.99	9.9	
	Median		7.99	7.74	
Weight	N		124	133	.630
	Mean		77.47	76.24	
	Median		72.85	74.7	
Number of medications on admission	N		131	131	**.019**
	Mean		10.66	12.06	
	Median		10	12	
PNI	N		112	116	.987
	Mean		42.57	42.49	
	Median		39.28	38.63	

Abbreviations: BMI, body mass index; ECOG, Eastern Cooperative Oncology Group; NLR, neutrophilto‐lymphocyte ratio; PNI, prognostic nutrition index; PLR, platelet‐to‐lymphocyte ratio; SII, systemic immune‐inflammation index; WBC, white cell blood count.

Bold values significance *P* values <0.05.

* The parametric *P*‐value is calculated by ANOVA for numerical covariates and chi‐square test for categorical covariates.

**Figure 1:**
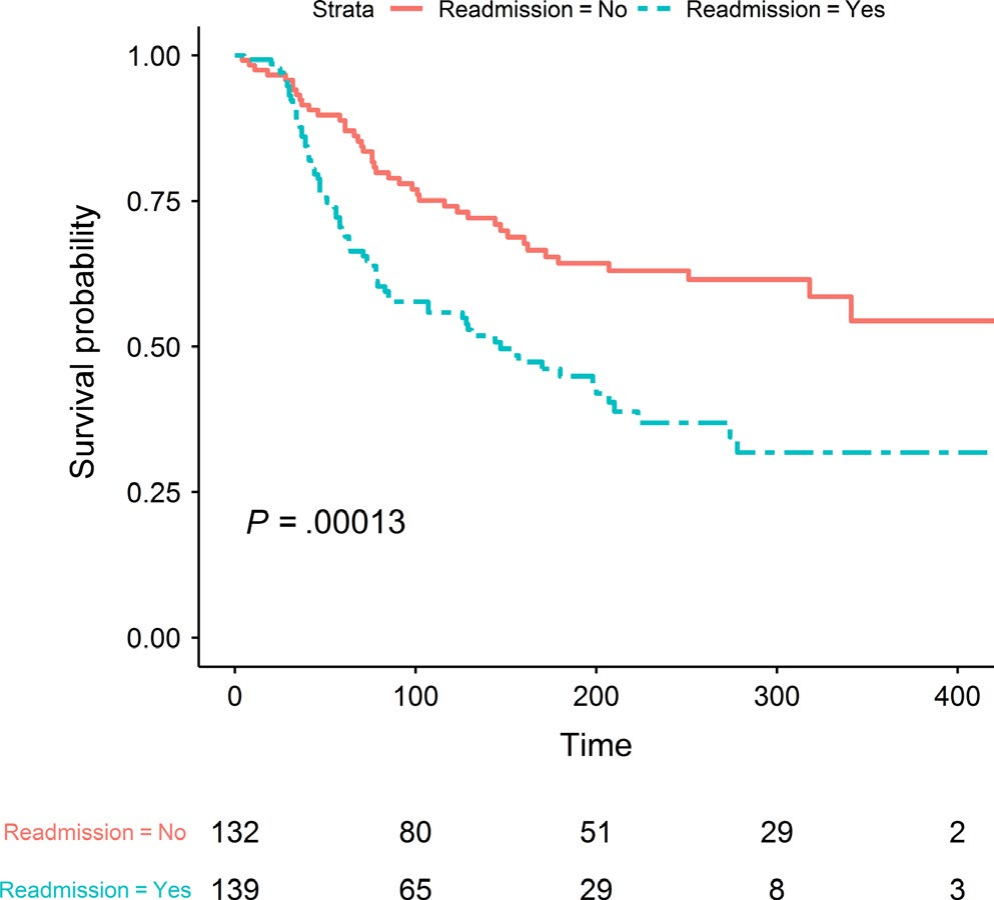
Kaplan‐Meier survival curves for overall survival according to 30‐d readmission category for 271 patients

A 30‐day readmission event was strongly associated with survival, with an adjusted hazard ratio (HR) of 2.39% and a 95% confidence interval (CI) of 1.46‐3.92. Patients who were readmitted within 30 days of discharge from their index admission had a median survival of 147 days compared with patients not readmitted who had not reached median survival by the end of the study (*P* < .0001) (Figure 1). In addition to 30‐day readmission, other significant predictors of survival identified in univariate Cox regression analyses included ECOG PS, metastasis, corrected calcium, length of index admission, inflammation indices (NLR, PLR, and SII), PNI, and leukocytosis (WBCs) (Table 4). Also notable, certain reasons for index admission (chemotherapy side effect, dehydration, neurological complaints, and “other” conditions); and two types of cancer (bladder and hepatocellular) were statistically significant predictors of survival. In the final multivariable Cox model, factors remaining statistically significant were 30‐day readmission event (HR = 2.44; 95% CI = 1.44‐4.12), corrected calcium, PNI, and leukocytosis (WBCs) (Table 5). To test whether missing data biased our results, we created 20 datasets with complete data using multiple imputations to estimate missing values. We tested these 20 datasets on the final multivariate Cox regression model. The range of the hazard ratios for readmission using these 20 tests was: 1.8‐2.0. All were statistically significant at the *P* ≤ .01 level.

**Table 4:** Univariate survival analysis

Covariate	Level	N	Hazard Ratio (95% CI)	HR *P*‐value
Sex	Female	132	0.99 (0.69‐1.43)	.971
	Male	139	—	—
Marital status	Divorced	28	0.87 (0.47‐1.60)	.644
	Single	35	0.71 (0.39‐1.31)	.272
	Widow	22	1.33 (0.72‐2.45)	.361
	Married	180	—	—
Language	Not English	11	1.12 (0.41‐3.03)	.830
	English	260	—	—
Race	Not White	38	1.05 (0.61‐1.82)	.850
	White	233	—	—
Categorized morse fall risk	Low	2	0.00 (0.00‐.)	.983
	Moderate	150	0.71 (0.49‐1.03)	.074
	Universal	10	0.14 (0.02‐1.04)	.055
	High	105	—	—
ECOG	1	109	1.37 (0.64‐2.94)	.413
	2	28	2.15 (0.89‐5.19)	.088
	3	15	2.54 (0.99‐6.49)	.052
	0	23	—	—
Reason for admission	Abnormal EKG/Lab/Image	26	0.64 (0.32‐1.27)	.201
	Chemotherapy Side Effect	30	0.48 (0.23‐0.98)	**.045**
	Dehydration	11	0.21 (0.05‐0.89)	**.035**
	Dyspnea	41	0.98 (0.57‐1.69)	.937
	Fever	21	0.54 (0.25‐1.18)	.123
	Neurological Complaint	8	0.13 (0.02‐0.93)	**.042**
	Other	78	0.59 (0.35‐0.99)	**.044**
	Pain	56	—	—
Cancer type	Bladder	9	3.96 (1.46‐10.74)	**.007**
	Breast	16	0.84 (0.33‐2.09)	.702
	GI	62	1.17 (0.66‐2.09)	.584
	Head and neck	13	1.42 (0.56‐3.55)	.459
	Hepatocellular	7	2.86 (1.14‐7.21)	**.025**
	Melanoma	22	0.80 (0.35‐1.83)	.590
	Neuroendocrine	8	0.26 (0.03‐1.93)	.187
	Other	76	0.89 (0.50‐1.59)	.697
	Sarcoma	14	0.75 (0.26‐2.22)	.608
	Lung	44	—	—
Histology category	Melanoma	22	0.80 (0.39‐1.65)	.548
	Other	29	1.02 (0.57‐1.83)	.943
	Sarcoma	22	0.71 (0.33‐1.54)	.385
	Carcinoma	197	—	—
Dasi	Moderate	139	1.88 (1.00‐3.52)	**.049**
	High	84	4.71 (2.48‐8.95)	**<.001**
	Low	48	—	—
Metastasis	Yes	159	1.79 (1.19‐2.70)	**.005**
	No	103	—	—
Readmission (Y/N)	Yes	139	2.08 (1.42‐3.05)	**<.001**
	No	132	—	—
Age		271	1.01 (0.99‐1.02)	.286
Health care visits previous 6 months		271	1.06 (0.89‐1.25)	.532
SCMC visits previous 6 months		271	0.94 (0.78‐1.13)	.514
Weight		265	0.99 (0.98‐1.00)	.154
Corrected calcium (Log transformed)		244	145.28 (23.09‐914.27)	**<**.**001**
Creatinine (Log Transformed)		255	0.74 (0.31‐1.75)	.487
Days between current admission and index admission		139	1.00 (0.97‐1.03)	.829
ECOG		175	1.39 (1.07‐1.82)	**.015**
Karnofsky		151	0.99 (0.97‐1.01)	.507
Height(cm)		240	1.00 (0.98‐1.02)	.679
Hemoglobin		267	1.02 (0.94‐1.11)	.628
Length of index admission(days)(Log Transformed)		271	1.33 (1.02‐1.73)	**.034**
Lymphocytes		254	0.86 (0.63‐1.19)	.359
NLR (Log transformed)		226	1.71 (1.36‐2.16)	**<**.**001**
Neutrophil (Log transformed)		227	2.41 (1.66‐3.50)	**<**.**001**
PLR (Log transformed		252	1.34 (1.08‐1.67)	**.008**
Platelet count (Log transformed)		267	1.58 (1.15‐2.15)	**.004**
SII (Log transformed)		225	1.65 (1.36‐2.00)	**<**.**001**
Albumin		248	0.53 (0.40‐0.72)	**<**.**001**
PNI		234	0.95 (0.93‐0.98)	**<**.**001**
Sodium		254	0.99 (0.95‐1.03)	0.503
WBC (Log transformed)		267	1.45 (1.13‐1.85)	**.004**
Weight		265	0.99 (0.98‐1.00)	.154
Number of diagnoses (Log transformed)		265	0.99 (0.98‐1.00)	.154
Number of medications on admission		267	1.01 (0.97‐1.05)	.807

Abbreviations: BMI, body mass index; ECOG, Eastern Cooperative Oncology Group; NLR, neutrophilto‐lymphocyte ratio; PNI, prognostic nutrition index; PLR, platelet‐to‐lymphocyte ratio; SII, systemic immune‐inflammation index; WBC, white cell blood count.

Bold values significance *P* values <0.05.

**Table 5:** Multivariable survival analysis

Covariate	Level	Hazard ratio	HR *P*‐value
Readmission (Y/N)	Yes	2.44 (1.44‐4.12)	**<.001**
	No	—	—
Health care visits previous 6 months		0.95 (0.75‐1.20)	.672
Cancer type	Bladder	2.79 (0.69‐11.30)	.150
	Breast	1.03 (0.34‐3.07)	.961
	GI	1.27 (0.58‐2.80)	.553
	Head and Neck	2.22 (0.64‐7.66)	.207
	Hepatocellular	4.74 (0.42‐53.14)	.207
	Melanoma	0.65 (0.26‐1.65)	.366
	Neuroendocrine	0.47 (0.06‐3.96)	.486
	Other	1.46 (0.66‐3.24)	.351
	Sarcoma	0.93 (0.26‐3.29)	.914
	Lung	—	—
Metastasis	Yes	1.35 (0.79‐2.30)	.266
	No	—	—
Reason for admission	Abnormal EKG/Lab/Image	0.75 (0.27‐2.09)	.580
	Chemotherapy side effect	1.08 (0.46‐2.53)	.859
	Dehydration	0.32 (0.07‐1.56)	.160
	Dyspnea	0.94 (0.42‐2.12)	.887
	Fever	0.47 (0.16‐1.41)	.179
	Neurological complaint	0.61 (0.08‐4.93)	.647
	Other	0.80 (0.41‐1.55)	.508
	Pain	—	—
Corrected calcium		1.55 (1.16‐2.06)	**.003**
Length of index admission(days)(Log transformed)		0.87 (0.60‐1.26)	.464
NLR (Log transformed)		0.83 (0.54‐1.30)	.423
PNI		0.94 (0.90‐0.99)	**.013**
WBC (Log transformed)		2.21 (1.18‐4.12)	**.013**

**Figure 2:**
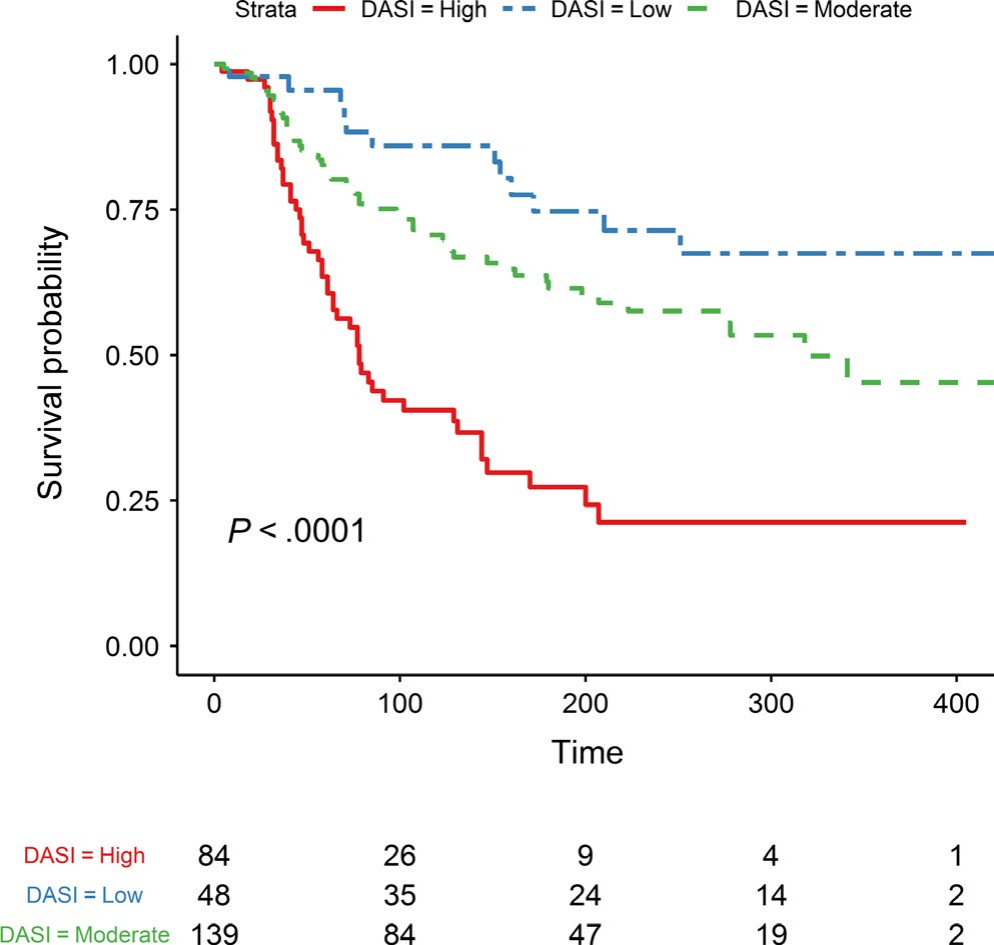
Kaplan‐Meier survival curves for overall survival in 271 patients

The DASI was a prominent predictor of survival. Relative to results in the low‐score group, hazard ratios in the moderate‐ and high‐score groups were 1.88 (95% CI, 1.00‐3.53; *P* = .049) and 4.71 (95% CI, 2.48‐8.95; *P* = .0001). The median survival time was 78 days (95% CI, 61‐131 days) for the high‐score group (cutoff value > 0.48), 318 days (95% CI, 207‐426 days) for the moderate‐score group (cutoff value, 0.31‐0.48), and not reached as of 426 days (*P* < .001) for the low‐score group (cutoff value < 0.31) (Figure 2).

## 4 DISCUSSION

Providing survival estimates is important for decision making in oncology care. Our anecdotal experience tells us that an unplanned acute hospitalization can signify a deterioration of performance status, often indicating a serious worsening of prognosis. However, being able to quantify this change in prognosis objectively has remained elusive. Our methodologic approach to estimate survival at the time of unplanned hospitalization and 30‐day readmission supports our experience. For patients with unplanned hospitalization followed by readmission within 30 days, median survival was 147 days. This worsening of survival in readmitted patients supports the use of readmission as a signal of the transition from the progressive to the terminal cancer phase.

For patients in our study group, factors associated with survival were not cancer specific, nor were they specific to the admitting diagnosis at the time of hospitalization, when combined with readmission in a multivariable statistical model. Rather, they represented general features common to the terminal cancer patient, such as widespread inflammation, malnutrition, biological disarrangements (such as hypercalcemia and leukocytosis). Correlations observed between NLR, PLR, and SII, but not PNI, suggested that the different inflammation indices were measuring the same thing in our study and suggested that PNI might be measuring a separate factor (malnutrition or cachexia). The identification of common features across cancer types supports the notion that the course of most cancers eventuates in a fairly universal clinical picture.

As a further refinement to our survival estimation, our formulated DASI system was found to be highly effective in predicting survival. The high‐score group had a median survival of 78 days vs >426 days in the low‐score group. Identifying patients who fall into the high‐score category will allow us to have a robust and timely discussion about end‐of‐life planning without having patients undergo unnecessary and costly medical interventions. To our knowledge, this is the first time this approach has been applied to cancer patients at the time of unplanned hospitalization. Our next step will be to prospectively validate this approach with attention to limitations related to existing clinical documentation.

Although our cohort mostly comprised patients with gastrointestinal and lung cancers and those having pain and dyspnea as their chief complaint, these factors were not statistically significant drivers of survival in our study. This is consistent with observations that quality of life indicators (including nausea and emesis, dyspnea, pain, and weakness) provide prognostic clues in patients with terminal cancer. Symptoms of dyspnea and pain may reflect progress of patients toward this terminal syndrome.^41^

A significant limitation is that our data were limited to those available in the electronic medical record. In certain instances, we used existing data such as nursing documentation of fall risk as a proxy for debility/robustness of the individual. We recognize that a true objective measure would be more informative, such as the validated Timed Up and Go study.^42^ We utilized “marital status” as a proxy for social support in lieu of the validated Social Support Questionnaire.^43^ We also recognize that we did not have adequate data on comorbidities and that implementation of tools such as the Charlson Comorbidity Index^44^ would be helpful. Hence, the fact that these variables were not significant in our analysis could be more a function of the limitations of our available data.

Although ECOG PS has long been accepted as a strong predictor of survival in cancer patients and was a significant predictor in our univariate analysis, our ECOG PS data may perhaps be not generalizable. The ECOG PS data were missing in 36% (186/287) of our cohort and 76% (142/186) had a documented ECOG PS of 1 or 0. The low ECOG PS may not be representative of the true clinical status of patients at the time of unplanned hospitalization. Because of lack of good quality data, we omitted ECOG PS from the multivariable analysis.

This study focused on patients receiving care at our free‐standing oncology center, resulting in limitations related to a lack of diversity among patients and lack of data for health care utilization at other sites. We recognize that further study is required to include cancer patients receiving care beyond our institution and service area.

This study represents a significant advancement in the methodology for assessment of survival at the time of unplanned hospitalization. We found that unplanned hospitalization followed by a 30‐day readmission event and our DASI system were important predictors of survival. The identified deficits found to be significant represent features common to the terminal cancer patient, such as widespread inflammation, malnutrition, biological disarrangements, and increased health care utilization. Utilization of a 30‐day readmission event and the DASI system could improve our current means of survival estimation in cancer patients with unplanned hospitalization. Future work will focus on prospective validation of our model with attention to overcome the limitations described above.

## RESEARCH SUPPORT

This work has been supported in part by the Shared Resources at the H. Lee Moffitt Cancer Center & Research Institute, an NCI designated Comprehensive Cancer Center (P30‐CA076292).

## PREVIOUS PRESENTATION

Contents of the manuscript have not been presented previously.

## CONFLICTS OF INTEREST

None.

## Data Availability

The data that support the findings of this study are available from the corresponding author upon reasonable request.
